# Reduced CX3CL1 Secretion Contributes to the Susceptibility of Oral Leukoplakia-Associated Fibroblasts to *Candida albicans*

**DOI:** 10.3389/fcimb.2016.00150

**Published:** 2016-11-11

**Authors:** Ran Cheng, Duo Li, Xueke Shi, Qinghong Gao, Changlei Wei, Xiaoyu Li, Yan Li, Hongmei Zhou

**Affiliations:** ^1^State Key Laboratory of Oral Diseases, Department of Preventive Dentistry, West China Hospital of Stomatology, Sichuan UniversityChengdu, China; ^2^State Key Laboratory of Oral Diseases, Department of Oral Medicine, West China Hospital of Stomatology, Sichuan UniversityChengdu, China; ^3^Department of Oral Medicine, School of Stomatology, Dalian Medical UniversityDalian, China; ^4^Department of Oral and Maxillofacial Surgery, West China Hospital of Stomatology, Sichuan UniversityChengdu, China; ^5^State Key Laboratory of Oral Diseases, West China Hospital of Stomatology, Sichuan UniversityChengdu, China

**Keywords:** antifungal peptide, *Candida albicans*, co-culture models, CX3CL1, fibroblast, leukoplakia

## Abstract

Candida leukoplakia (OLK) is a kind of oral leukoplakia combined with chronic candidal infection, which plays an important role in the malignant transformation of OLK. However, little is known about the etiology, including susceptibility of leukoplakia to candidal adhesion, invasion and infection. Some antimicrobial peptides secreted by oral epithelial cells or fibroblasts potentially have antifungal activities against *Candida albicans (C. albicans)*. In this study, we established three co-culture models to simulate different *C. albican*s-fibroblasts interactions during progression of candida leukoplakia. The susceptibility of oral leukoplakia-associated fibroblasts (LKAFs) to *C. albicans* and its underlying mechanism were determined. Samples of 14 LKAFs and 10 normal fibroblasts (NFs) were collected. The co-culture models showed that LKAFs had promoted the adhesion, invasion, and survival of *C. albicans* compared with NFs. CX3CL1, a chemokine with antifungal activity, was less abundant in LKAFs than NFs. Overexpression of CX3CL1 via transfection in LKAFs could partly restore the resistance to *C. albicans*. We also showed that inhibition of ERK could suppress CX3CL1 secretion. While phosphor-ERK was inhibited in LKAFs compared with NFs. Besides, the mRNA expression of a shedding enzyme for CX3CL1, disintegrin and metalloproteinase domain (ADAM) 17 was decreased in LKAFs than NFs. In conclusion, LKAFs produced and secreted less CX3CL1 by inhibiting the ERK signaling pathway, thereby contributing to impaired cell resistance to *C. albicans*.

## Introduction

Oral leukoplakia (OLK) is one of the most common potentially malignant oral disorders. The location, duration, and type of OLK, smoking habit, and candidal infection are risk factors for malignant transformation of OLK (Yardimci et al., 2014). *Candida albicans* is the predominant fungal species associated with OLK (Krogh et al., 1987) and it has great potential to induce candidiasis. The term “candida leukoplakia” is used to describe leukoplakia combined with chronic candidal infection, which plays an important role in the malignant transformation of OLK (Lehner, 1964).

*C. albicans* is the most common *Candida* species in the oral cavity where it is a commensal fungus in healthy individuals (Odds, 1988). Systemic factors such as severe nutritional deficiency and generalized immune suppression play contributory roles in candidal infections. However, the occurrence of these systemic disorders is not very high in cases of candida leukoplakia (Sitheeque and Samaranayake, 2003). Thus, local factors related to OLK are considered to be important. Some cases of leukoplakia occur along the occlusal line and it is hypothesized that continuous occlusal stress or trauma contributes to candida leukoplakia (Walker and Arendorf, 1990). Epithelial disorders in OLK, such as atrophy, hyperplasia, and dysplasia, may compromise the mucosal barrier and facilitate candida invasion as well (Samaranayake, 1990).

The oral mucosa comprises the epithelium and stroma, providing the initial physical defense against infection. Epithelial mesenchymal interactions (EMIs) are known to be essential for cell growth, differentiation and tumorigenesis. In the oral cavity, EMIs are observed in odontogenesis, dentino-enamel junction formation, and carcinogenic processes (Santosh and Jones, 2014). It can be assumed that epithelial dysplasia, as well as altered biological characteristics of the adjacent stroma (fibroblasts) facilitate candidal infection in candida leukoplakia.

Two important antifungal mechanisms are found in healthy individuals. First, oral epithelial cells and fibroblasts have important roles in the natural host defense against *C. albicans*. Oral epithelial cells provide a physical barrier against *C. albicans* and they respond to invading *Candida* species by producing antimicrobial peptides. Oral epithelial cells have been reported to produce β-defensin (Zasloff, 1992; Ouhara et al., 2005), which is effective at killing *C. albicans*. Oral fibroblasts are able to secret CX3CL1, a chemokine with antifungal activity against *C. albicans* (Ohta et al., 2010). The second antifungal defense mechanism is the inflammatory responses. *C. albicans* trigger epithelial cells and increase the production of interleukin (IL)-1β, IL-6 and IL-23 (Feller et al., 2014). Besides, the exposure of fibroblasts to *C. albicans* also leads to the release of IL-8 and IL-6 (Dongari-Bagtzoglou et al., 1999). These pro-inflammatory factors recruit neutrophils and macrophages to the site of the infection which contributes to exacerbating inflammation.

In most cases, *C. albicans* do not induce oral candidiasis in healthy individuals. The host and *C. albicans* maintain an ecological equilibrium. In general, it is considered that self-defense by epithelial cells and fibroblasts is greatly important for host-pathogen interactions. Most researches have focused on the roles of the epithelial barrier and epithelial cells in defending against Candida (Moyes et al., 2010; Rouabhia et al., 2012; Hua et al., 2014). However, fibroblast-containing connective tissue is also involved in the molecular pathogenesis of oral candidiasis (Yadev et al., 2011). The epithelial dysplasia, abnormal stoma and EMIs of OLK could be a pathological factor of candida leukoplakia. Thus, we assume that OLK-associated fibroblasts (LKAFs) may be defective in terms of candidal resistance. There is currently no information about the role of fibroblasts in candida leukoplakia. In this study, we investigated the susceptibility of LKAFs to *C. albicans* and we aimed to elucidate possible mechanisms.

## Materials and methods

### Tissue collection

OLK tissues obtained from surgical resection and normal oral tissues obtained from plastic surgery were collected at West China Hospital of Stomatology, Sichuan University (Chengdu, Sichuan, China). Clinical and pathological diagnoses were made according to the criteria for OLK (Reibel, 2003). Patients were eligible for inclusion if they were: (1) aged between 18 and 75 years, and (2) diagnosed with OLK. Patients were excluded if they had: (1) severe candidal infection, (2) severe systemic diseases, (3) received antibiotics or other medicines in the previous 4 weeks, (4) other oral mucosal diseases, and (5) complete or removable partial dentures. In total, 17 OLK tissue samples, two oral squamous cell carcinoma (OSCC) tissue samples, and 10 normal tissue samples were collected. The patient information of OLK and OSCC was shown in Supplement Table 1. The protocol was reviewed and approved by the Institutional Ethics Committee of West China Hospital of Stomatology (WCHSIRB-D2012-037). All subjects provided their informed written consent.

### Cell culture and immunocytochemistry

To obtain LKAFs, carcinoma-associated fibroblasts (CAFs), and normal fibroblasts (NFs), the tissues were isolated, cultured, and purified using previously described protocols (Liu et al., 2006; Meng et al., 2014). The collected tissues were cut into pieces and cultured in Dulbecco's modified Eagle's medium (DMEM; GIBCO, Thermo Fisher Scientific Inc., Waltham, MA, USA) with 10% fetal bovine serum (GIBCO) at 37°C with 5% CO_2_. A human oral squamous cell carcinoma cell (OSCC) line was used to simulate severe dysplasia epithelial cells. OSCC cell line has been used to study interaction between *C. albicans* and epithelial cells (Moyes et al., 2010). In our study, HSC-2(American Type Culture Collection, ATCC, Manassas, VA, USA) was chosen and cultured in the same condition as primary fibroblast cells.

In total, 17 LKAFs, 10 NFs, and two CAFs were seeded on glass coverslips in six-well plates. Immunocytochemical (ICC) staining was performed as described in the manufacturers' protocols. The primary antibodies were anti-cytokeratin (1:200; ZSGB-BIO, Beijing, China), anti-vimentin (1:200; ZSGB-BIO), anti-α-smooth muscle actin (α-SMA, 1:1000; Abcam, Cambridge, UK) and anti-fibroblast activation protein (FAP, 1:1000; Abcam). The immunostained cells were examined in at least five randomly selected visual fields under a light microscope (Nikon Eclipse 80i, Tokyo, Japan). The obtained images were compiled using Adobe Photoshop CS6 software (Adobe Systems Inc., San Jose, CA, USA). The ICC scores were determined as described previously (Kreisberg et al., 2004). Subsequently, 14 LKAFs and 10 NFs were used in the following experiments. The fibroblasts were harvested and counted with a hemocytometer (Qiujing, Shanghai, China) before use.

### *C. albicans'* growth

*C. albicans* ATCC 10231 was obtained from the State Key Laboratory of Oral Science, West China Hospital of Stomatology. The yeast cells were cultured for 48 h on Sabouraud's agar plates (tryptone 10 g, glucose 40 g, and agarose 20 g in 1000 mL H_2_O; pH 5.6 ± 0.2) at 37°C. The *C. albicans* were then harvested, washed three times with phosphate-buffered saline (PBS) and counted with nephelometer (Becton, Dickinson and Company Franklin Lakes, NJ, USA) before use.

### Construction of lentiviral vectors and transfection

Lentiviral vector GV287 tagged with enhanced green fluorescence protein was used. The vector with the human *CX3CL1* gene (NM_002996) and vector with a control sequence were constructed by Genechem Inc. (Shanghai, China). The LKAFs (10^4^ cells/well) were seeded in 96-well plates overnight before transfection. The virus (at a multiplicity of infection (MOI) of 10) was added to each well containing polybrene (5 μg/mL) (Genechem Inc.) and incubated for 8 h at 37°C. After transfection, cells were incubated in complete medium for 72 h. Samples of the cells and supernatant were collected for real-time PCR, western blotting, and enzyme-linked immunosorbent assay (ELISA).

### Scanning electron microscopy (SEM)

*C. albicans* (MOI = 1) were directly co-cultured with 2 × 10^6^ LKAFs or NFs for 2 and 4 h. The samples were rinsed with distilled water and fixed overnight in 3% glutaraldehyde at 4°C. Dehydration was accomplished using the following ethanol gradient: 30, 50, 70, 95, and 100%. The samples were then dehydrated with xylene and air-dried at room temperature. Subsequently, the specimens were coated with gold and examined by SEM (HITACHI-S-3400N, Hitachi High-Technologies Corporation, Tokyo, Japan). The obtained images were processed using Adobe Photoshop CS6 software (Adobe Systems Inc., San Jose, CA, USA).

### *C. albicans* adhesion assay and invasion assay

For the adhesion assay and invasion assay, the co-culturing time were 2 h (adhesion) (Moyes et al., 2010) and 4 h (invasion), respectively.

#### Supernatant indirect culture

*C. albicans*, epithelial cells and fibroblasts supernatant (non-stimulated fibroblasts) were co-cultured. 2 × 10^6^ LKAFs or NFs were kept in serum-free DMEM in six-well plates for 24 h. Then the supernatant was collected and incubated with *C. albicans* (MOI = 1) for 24 h. Next, the supernatant containing *C. albicans* was co-cultured with HSC-2 cells for 2 and 4 h.

#### Transwell indirect culture

*C. albicans*, epithelial cells and fibroblasts (indirectly stimulated fibroblasts) were co-cultured. 2 × 10^6^ LKAFs or NFs were seeded in the upper chamber of transwell (0.4 μm; Corning Inc., NY, USA) in 500 μL serum-free media. In addition, 2 × 10^6^ HSC-2 cells and *C. albicans* (MOI = 1) were incubated in the lower chamber in 500 μL serum-free media for 2 and 4 h.

#### Direct culture

*C. albicans* and fibroblasts (directly stimulated fibroblasts) were co-cultured. 2 × 10^6^ LKAFs or NFs were incubated with *C. albicans* (MOI = 1) in serum-free media in six-well plates for 2 and 4 h.

For the adhesion assay, the supernatant samples containing *C. albicans* (unattached yeasts) were collected. Loosely adherent *C. albicans* were softly washed by PBS once and collected. These unattached *C. albicans* were centrifuged and resuspended. 100 μL suspension was incubated on Sabouraud's agar plates for 24 h. Colonies per plate were numerated by colony counter (Synoptics Ltd., Cambridge, UK).

For the invasion assay, the supernatant samples containing *C. albicans* (unattached yeasts) were collected. Adherent but not invasive *C. albicans* were strongly washed by PBS for three times and collected. The collected *C. albicans* were assessed the same as the adhesion assay.

To determine total number of *C. albicans*, a control group was designed, with the same amount of *C. albicans* and the same culture condition, but without fibroblasts. The *C. albicans* were scraped and collected. The assessment was the same as the adhesion assay. The number of attached *C. albicans* was calculated as: total number—unattached number. The adhesive/invasive rate was calculated as: (total number—unattached number)/total number × 100%.

### *C. albicans* survival assay

#### Supernatant indirect culture

*C. albicans* and fibroblasts supernatant (non-stimulated fibroblasts) were co-cultured. 2 × 10^6^ LKAFs or NFs were cultured in serum-free media for 24 h in six-well plates. The supernatant was collected and incubated with *C. albicans* (MOI from 10^−2^ to 10^−5^) for 24 h.

#### Transwell indirect culture

*C. albicans* and fibroblasts (indirectly stimulated fibroblasts) were co-cultured. 2 × 10^6^ LKAFs or NFs were seeded in the lower chamber in 500 μL serum-free media. In addition, 500 μL of *C. albicans* suspension (MOI from 10^−2^ to 10^−5^) was incubated in the upper chamber for 24 h.

#### Direct culture

*C. albicans* and fibroblasts (directly stimulated fibroblasts) were co-cultured. 2 × 10^6^ LKAFs or NFs were incubated with *C. albicans* (MOI from 10^−2^ to 10^−5^) in serum-free media in six-well plates for 24 h.

After direct or indirect culture, the supernatant samples containing *C. albicans* were collected and incubated in Sabouraud's agar plates for 24 h at 37°C. The amount of *C. albicans* was assessed by colony-forming units (CFU)/mL and were recorded as log[CFU/mL].

### Inhibition of signaling pathways

To test the involvement of candidate signaling pathways, the specific inhibitors, U0126 of ERK MAPK, SB203580 for p38 MAPK, SP600125 for JNK MAPK and BAY11-7082 for NFκB were obtained from Beyotime Inc., Shanghai, China. DMSO were obtained from Sigma-Aldrich, St. Louis., MO, USA.

5 × 10^4^ NFs were seeded in 6-well plates until 80–90% confluence. Then the NFs were pretreated with 20 μM U0126, 50 μM SB203580, 100 μM SP600125 or 50 uM BAY11-7082 in 1 mL serum-free media for 4 h, respectively. Later, 1 × 10^5^ CFU/mL *C. albicans* were co-cultured with NFs and inhibitors for another 4 h. The supernatants were collected for ELISA.

### Real-time PCR

1 × 10^5^ CFU/mL *C. albicans* and 5 × 10^4^ NFs or LKAFs were co-cultured in 1 mL of serum-free media in 6-well plates. After 0 or 4 h, the supernatant samples and cells were collected to detect CX3CL1 mRNA expression and secretion by real-time PCR or ELISA, respectively.

Total mRNA was isolated using TRIzol (Invitrogen; Thermo Fisher Scientific Inc., Waltham, MA, USA) and 1 μg of the total RNA was used to synthesize cDNA with a PrimeScript RT reagent Kit (Perfect Real Time, Takara, Dalian, China). The mRNA expression levels were determined by real-time PCR using a PRISM 7300 Sequence Detection System PCR (Applied Biosystems, Thermo Fisher Scientific Inc., Waltham, MA, USA) with SYBR Premix DimerEraser (Perfect Real Time, Takara). The primer sequences used were shown in Supplement Table 2.

### ELISA assay

The levels of CX3CL1 in the culture supernatants were determined by ELISA (USCN Life Science Inc., Wuhan, China), according to the manufacturer's instructions.

### Western blotting

Western blotting was performed as described previously (Meng et al., 2014). Cells were lysed in lysis buffer (Keygen Biotech Inc., Nanjing, China) and 30 μg of the total proteins were loaded for each sample. The primary antibodies were anti-CX3CL1 (1:2000; Abcam), anti-ERK1/2 (1:2000; Cell Signaling Technology Inc.), anti-phospho-ERK1/2 (1:2000; Cell Signaling Technology Inc.), and anti-GAPDH (1:2000; Abcam). After incubating with the secondary antibody (Santa Crus Biotechnology, Inc., CA, USA), the proteins were visualized using an ECL kit obtained from PerkinElmer (Millipore Inc., Darmstadt, Germany).

### Statistical analysis

The results were expressed as the mean ± SD based on 3-4 separate experiments. ANOVA and Student's *t*-test were used for statistical analyses. *P* < 0.05 was considered to indicate a statistically significant difference.

## Results

### LKAFS gained some features of cancer-associated fibroblasts (CAFs)

**Figure 1:**
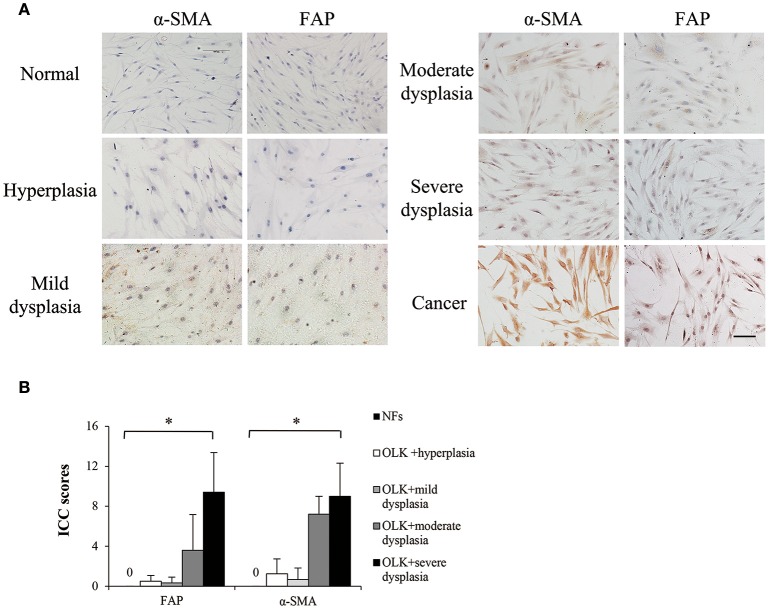
**Markers of carcinoma-associated fibroblasts (CAFs) expressed in LKAFs. (A)** Immunostaining of α-SMA and FAP in NFs (*n* = 10), LKAFs (*n* = 17), and CAFs (positive control). Scale bar, 50 μm. **(B)** ICC scores for α-SMA and FAP in NFs and LKAFs with different epithelial dysplasia (ANOVA, ^*^P < 0.05).

Currently, it is in agreement that cancer activates resident fibroblasts, termed carcinoma-associated fibroblasts, which participate in neoplastic progression (Mao et al., 2013). α-smooth muscle actin (α-SMA) and fibroblast activation protein (FAP) are well-established markers of CAFs (Kalluri and Zeisberg, 2006). OLK is a premalignant lesion of the oral mucosa. Theoretically, LKAFs are able to undergo similar activation by OLK. But whether and which level LKAFs are activated remain unknown. In this study, we used immunostaining of α-SMA and FAP to examine the differences among NFs, LKAFs, and CAFs. The results showed that LKAFs exhibited weak and moderate positive staining for α-SMA and FAP, whereas CAFs had strong positive staining (Figure 1A). The ICC score for LKAFs increased when the epithelial dysplasia was greater (Figure 1B). The results confirmed that LKAFs were different from NFs. LKAFs had gradually expressed CAFs' markers when epithelia became dysplastic. Thus, LKAFs potentially influence initiation and progression of some specific disease, e.g., candidal infection. Our subsequent investigations attempted to prove the possibility.

### The establishment of co-culture models

Candida leukoplakia is characterized histologically by intraepithelial inflammation with fungal hyphae invading the superficial epithelial layer (Cawson and Lehner, 1968). *C. albicans* adhere and convert to the hyphal form which penetrates into the oral mucosa, causing tissue damage (Naglik et al., 2011). When the hyphae of *C. albicans* traverse the entire epithelium, further invasion to the stroma may happen (Reichart et al., 1995). Based these findings, three co-culture models were established to simulate the process when *C. albicans* invade different levels of oral mucosa.

#### Supernatant indirect culture: to simulate adhesive and colonizing process

During *C. albicans* adhesion and colonization, epithelial cells are the first line of defense and initiates innate and adaptive immune responses (Hebecker et al., 2014). But the role of stromal cells in candida leukoplakia is unknown. Here we simulate an early adhesive and colonizing process. *C. albicans* and fibroblasts supernatant (non-stimulated fibroblasts) were co-cultured, where non-stimulated secretary products of fibroblasts were involved. Then the adhesion, invasion and survival of *C. albicans* were examined.

#### Transwell indirect culture: to simulate *C. albicans* invasion in epithelial layer

After colonization, the attached *C. albicans* penetrate either directly into host cells or at intercellular junctions between cells, leading to host-microbe interaction (Hebecker et al., 2014). It is assumed that an indirect interaction between *C. albicans* and fibroblasts exists. Here *C. albicans* and fibroblasts were indirectly co-cultured by using transwell, where stimulated secretary products of *C. albicans* and fibroblasts were included. Then the adhesion, invasion and survival of *C. albicans* were examined.

#### Direct culture: to simulate *C. albicans* invasion in deeper layer

Later phases of *C. albicans* infection are characterized by damage and loss of the superficial epithelium due to invasion of long *C. albicans* hyphae (Hebecker et al., 2014). In specific case, hyphae may cross basal membrane and invade adjacent connective tissue (Reichart et al., 1995). It is assumed that *C. albicans* hyphae have more direct interaction with fibroblasts, where both stimulated secretary products and contacting mechanism were involved. Here *C. albicans* and fibroblasts were directly co-cultured, to examine the invasion and survival of *C. albicans*.

### LKAFs were more susceptible to *C. albicans* than NFs

**Figure 2:**
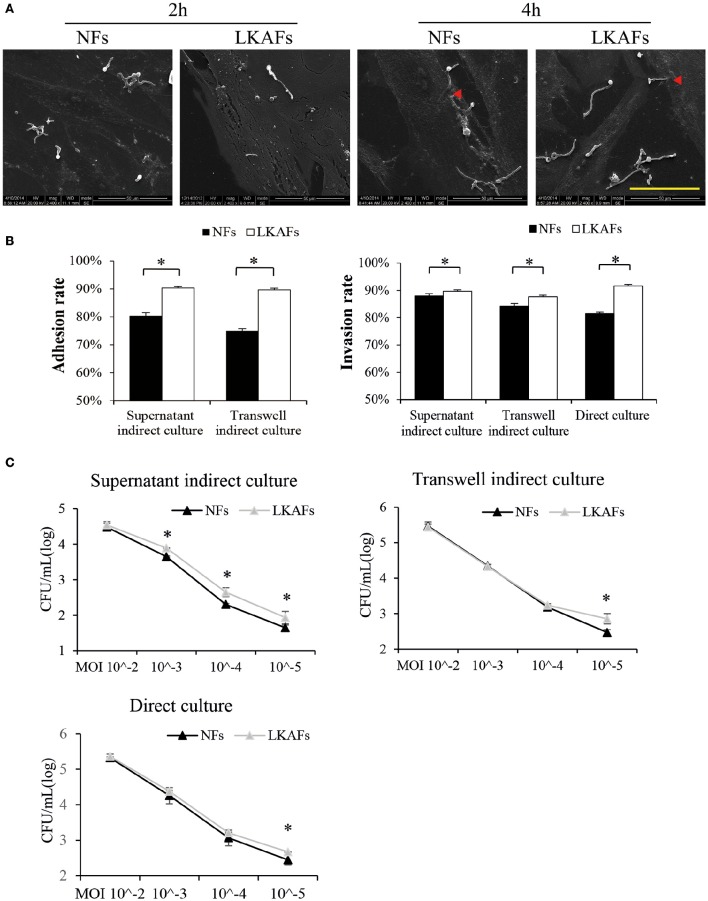
**NFs were more resistant to *C. albicans* than LKAFs. (A)** LKAFs or NFs and *C. albicans* were co-cultured directly for 2 and 4 h. *C. albicans* adhesion and invasion were observed by SEM. Scale bar, 50 μm. Red triangles represent invasion by *C. albicans*. The results showed that *C. albicans* started adhesion within 2 h and commenced invasion at 4 h. **(B)** The three co-culture models, supernatant indirect culture, transwell indirect culture, and direct culture were used to co-culture fibroblasts (four NFs and three LKAFs) and *C. albicans*. Co-culturing for 2 and 4 h were used in the adhesion assay and invasion assay. Data represent the mean percentage ± SD based on three independent experiments (independent samples *t*-test, ^*^P < 0.05). **(C)** Co-culture of fibroblasts (four NFs and three LKAFs) with *C. albicans* for 24 h. The surviving *C. albicans* in the supernatant were collected for further culture and colony counting. Data represent the mean log (CFU/mL) ± SD based on three independent experiments (independent samples *t*-test, ^*^P < 0.05).

The adhesion and invasion of *C. albicans* to tissue are essential for candidiasis (Hazen et al., 1991). We found *C. albicans* could adhere to both NFs and LKAFs within 2 h. Some hyphae growth was observed at 2 h. Incubation for 4 h resulted in more hyphae growth, which initiated invasion to fibroblasts (Figure 2A).

The three co-culture models were used to determine the susceptibility of LKAFs to *C. albicans*. *C. albicans* were more adhesive to HSC-2 when co-culturing with LKAFs than NFs. Thus, LKAFs group had higher *C. albicans* adhesive rate than NFs. Furthermore, indirect co-culture of LKAFs increased the invasion of *C. albicans* to HSC-2. *C. albicans* were also more invasive to LKAFs than NFs under direct culture conditions (Figure 2B). The results showed that LKAFs promoted adhesion and invasion of *C. albicans* in *three* types of *in vitro* co-culture models. LKAFs are potentially effective in promoting the initiation and progression of candida leukoplakia.

In the *C. albicans* survival experiment, supernatant indirect culture showed that LKAFs increased *C. albicans* survival ability than NFs when MOI was from 10^−3^ to 10^−5^. The results confirmed that fibroblasts played a role in host defense to *C. albicans* as early as adhesive and colonizing process. LKAFs had lost some antifungal ability compared with NFs. However, other co-culturing methods exhibited similar trend when MOI was as low as 10^−5^ (Figure 2C). Therefore, LKAFs may be less resistant than NFs only when limited *C. albicans* invaded. But the antifungal effect of NFs diminished when C. *albicans* survived and proliferated. The role of LKAFs in facilitating candida leukoplakia tends to be more obvious in the early stage.

Furthermore, both direct and indirect co-culture methods exhibited differences between LKAFs and NFs. There is a possibility that LKAFs lack some secretory antifungal mechanism to resist *C. albicans* compared with NFs, which resulted in indirect effects to *C. albicans*. Next, we tried to figure out the secretory factor.

### LKAFs produced and secreted less CX3CL1 than NFs

**Figure 3:**
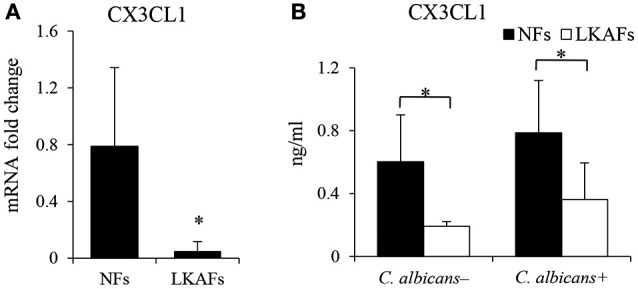
**NFs produced more CX3CL1 than LKAFs. (A)** LKAFs (*n* = 9) and NFs (*n* = 5) were exposed to *C. albicans* for 4 h and the mRNA levels of CX3CL1 were determined. Data represent the mean fold change ± SD based on three independent experiments (independent samples *t*-test, ^*^P < 0.05). **(B)** LKAFs (*n* = 5) and NFs (*n* = 8) were cultured with *C. albicans* for 0 and 4 h, and the supernatants were collected for ELISA. Data represent the mean concentration ± SD based on three independent experiments (independent samples *t*-test: ^*^P < 0.05).

CX3CL1, a potential antifungal cytokine, is considered as a candidate for the secretory antifungal mechanism. It is reported *C. albicans* infected oral fibroblasts are able to secret CX3CL1, a potential antifungal chemokine (Ohta et al., 2010). We tried to compare the CX3CL1 level in LKAFs and NFs when they were infected by *C. albicans*. As shown in Figure 3, the mRNA level of CX3CL1 in the LKAFs appeared to be lower than NFs. CX3CL1 secretion revealed the same trend in LKAFs, with or without *C. albicans* stimulation. Thus, CX3CL1 might be involved in the mechanism that mediates decreased resistance of LKAFs to *C. albicans*.

### LKAFs with overexpressed CX3CL1 partly regained resistance to *C. albicans*

**Figure 4:**
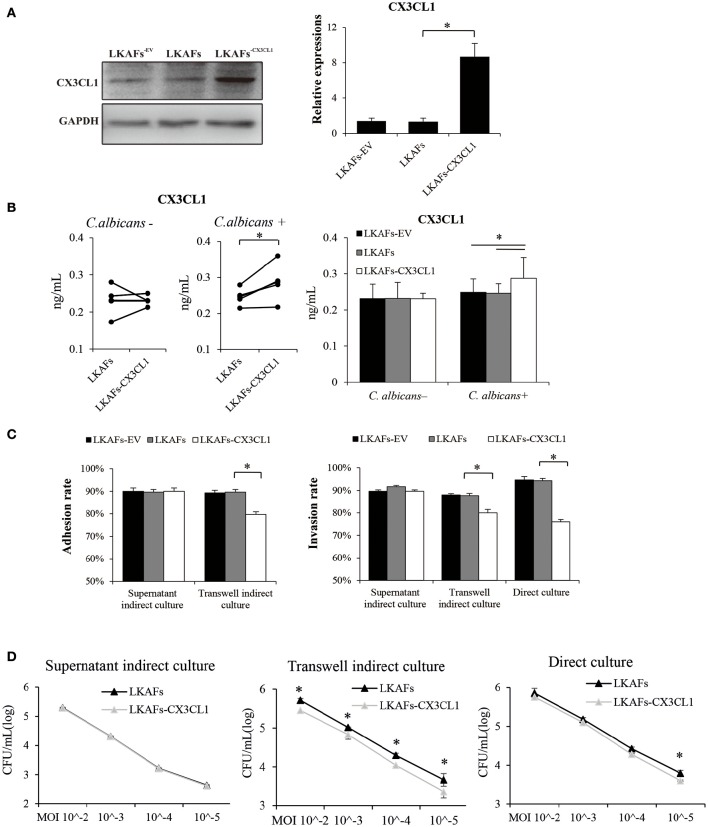
**Overexpression of CX3CL1 partially restored the resistance to *C. albicans* in LKAFs. (A)** A vector for overexpressing CX3CL1 was transfected into LKAFs (*n* = 4). After 48 h, western blotting analysis indicated that CX3CL1 was highly expressed in LKAFs^−*CX*3*CL*1^ cells (paired samples *t*-test, ^*^P < 0.05). **(B)** LKAFs^−*CX*3*CL*1^ cells and the control groups were co-cultured with *C. albicans* for 0 and 4 h. Non-stimulated LKAFs^−*CX*3*CL*1^ cells had no change in CX3CL1 secretion compared with the controls. Stimulation of *C. albicans* increased CX3CL1 secretion in LKAFs^−*CX*3*CL*1^ cells (paired samples *t*-test, ^*^P < 0.05). **(C)** The three co-culture models of fibroblasts (*n* = 4) and *C. albicans* for 2 h (adhesion assay) and 4 h (invasion assay). Data represent the mean percentage ± SD based on three independent experiments (^*^P < 0.05). **(D)** Co-culture of fibroblasts (*n* = 4) and *C. albicans* for 24 h. LKAFs^−*CX*3*CL*1^ cells exhibited lower survival of *C. albicans* in the supernatant compared with LKAFs. Data represent the mean log (CFU/mL) ± SD based on three independent experiments (paired samples *t*-test ^*^P < 0.05).

To examine whether CX3CL1 influenced the resistance of LKAFs to *C. albicans*, we transfected lentivirus vectors so that the protein level of CX3CL1 was highly increased (Figure 4A). However, CX3CL1 secretion was not highly promoted as we had expected. We found non-stimulated fibroblasts didn't increase CX3CL1 secretion (Figure 4B). As a result, supernatant indirect culture showed no difference between LKAFs and LKAFs^−*CX*3*CL*1^ cells in both adhesion assay and invasion assay. On the contrary, stimulated LKAFs^−*CX*3*CL*1^ cells had higher CX3CL1 secretion. Consequently, in transwell indirect culture and direct culture conditions where fibroblasts were stimulated, *C. albicans* had lower adhesive rate and invasive rate to LKAFs^−*CX*3*CL*1^ cells than LKAFs (Figure 4C). The survival assay showed the same trend (Figure 4D). Therefore, increased CX3CL1 secretion promoted resistant ability to *C. albicans*. CX3CL1 may be involved in anti-fungal mechanism of fibroblasts. However, CX3CL1 secretion is not merely influenced by protein production. Some other factors, e.g., sheddases, the enzymes for cleaving, may be more important.

### ERK MAPK and ADAM17 influenced CX3CL1 level

**Figure 5:**
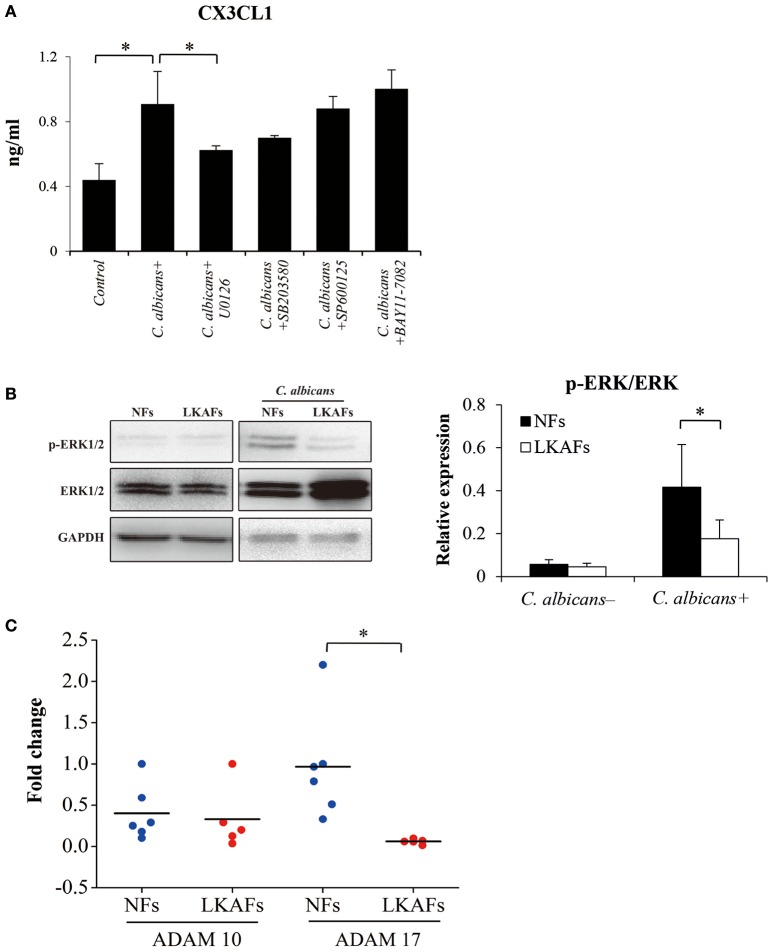
**Possible mechanism that allowed fibroblasts to mediate CX3CL1 secretion. (A)** NFs (*n* = 3) were pretreated with 20 μM U0126, 50 μM SB203580, 100 μM SP600125, and 50 μM BAY11-7082 for 4 h, and then stimulated by *C. albicans* for 4 h. The supernatants were collected for ELISA (ANOVA, ^*^P < 0.05). **(B)** To examine the activation of ERK MAPK, NFs (*n* = 5) and LKAFs (*n* = 5) were co-cultured with *C. albicans* for 0 and 2 h (^*^P < 0.05). **(C)** The mRNA levels of ADAM10 and ADAM17 in NFs (*n* = 5) and LKAFs (*n* = 6) were compared (independent samples *t*-test, ^*^P < 0.05).

We showed that CX3CL1 was an important cytokine that affects the antifungal ability of NFs and LKAFs. Later, we examined the relationships between CX3CL1 and signaling pathways, ERK MAPK, p38 MAPK, JNK MAPK, NF-κB, by using 20 μM U0126 as inhibitor of the ERK pathway, 50 μM SB203580 as inhibitor of p38 MAPK, 100 μM SP600125 as inhibitor of JNK MAPK, and 50 μM BAY11-7082 as inhibitor of NF-κB. LKAFs were not shown here for the CX3CL1 production has been impaired. We found that ERK MAPK influenced CX3CL1 secretion. Inhibition of ERK MAPK greatly reduced the secretion of CX3CL1 (Figure 5A). The phosphor-ERK1/2 (p-ERK) level verified that the activation of ERK in LKAFs was much lower than that in NFs with stimulation of *C. albicans*. This also confirmed our finding that ERK MAPK influenced CX3CL1 secretion (Figure 5B).

Various chemokines are associated with sheddase before secretion, where ADAM10 and ADAM17 are particularly involved in the shedding of CX3CL1 (Garton et al., 2001; Hundhausen et al., 2003). Our results showed that the mRNA level of ADAM17, but not ADAM10, was decreased in LKAFs compared with NFs (Figure 5C).

## Discussion

*C. albicans* is a normal commensal in the oral cavity of healthy individuals but it can be an opportunistic pathogen when the host defense is impaired (Odds, 1988). Thus, we hypothesized that OLK may be a possible promoter of candidal infection. LKAFs, which had obtained some feature of CAFs (Figure 1), may be involved in the process of candidal infection. In this study, we collected 14 leukoplakia fibroblast samples and 10 healthy fibroblast samples to elucidate the underlying mechanism related to candidal infection in OLK.

### LKAFs promoted adhesion, invasion, and survival of *C. albicans*

*C. albicans* adhesion to host cells is essential for increasing colonization by yeasts (Dalle et al., 2010). Then, hyphae-mediated invasion is critical in candidal infections (Hebecker et al., 2014). Here we established three co-culture models to simulate initiation and progression of candida leukoplakia. Supernatant indirect culture represented *C. albicans* adhesion and colonization. Transwell indirect culture represented *C. albicans* invasion in epithelial layer. Direct culture represented *C. albicans* hyphae invasion extended to stromal layer. By using the methods, we have found *C. albicans* adhesion and invasion were enhanced by LKAFs in all the stages, thereby supporting our hypothesis that the natural resistance of LKAFs to *C. albicans* is partially impaired.

We investigated the differences between LKAFs and NFs when they were under attack by *C. albicans*, where the results demonstrated that low dose *C. albicans* exhibited greater survival ability when co-cultured with LKAFs than NFs. But the differences disappeared when the amount of *C. albicans* increased. We suspected that LKAFs promote candida leukoplakia in the initiating process, especially when limited *C. albicans* start adhesion and colonization. Furthermore, the indirect cultures exhibited significant difference between LKAFs and NFs, indicating a secretory factor was involved (Figure 2). Thus, we suggested that NFs provide some cytokines against *C. albicans* during all the adhesion, invasion and infectious process, whereas the defenses are partially impaired in LKAFs.

### Decreased CX3CL1 secretion in LKAFs impaired its resistance to *C. albicans*

It has been reported that gingival fibroblasts can respond to *C. albicans* by secreting CX3CL1, which is considered to be an antifungal cytokine that affects the survival of *C. albicans* (Ohta et al., 2010). Therefore, CX3CL1 is regarded as a candidate in direct host defense against *C. albicans*. As expected, we found less CX3CL1 was secreted by LKAFs, with or without *C. albicans* stimulation (Figure 3). It verified our results that non-stimulated and stimulated LKAFs enhanced *C. albicans*' adhesion, invasion and survival. Later, we overexpressed CX3CL1 in LKAFs (Figure 4). However, LKAFs^−*CX*3*CL*1^ cells increased CX3CL1 secretion only after *C. albicans*' stimulation. Thus, no differences were found in supernatant indirect culture (non-stimulated fibroblasts). The results highly supported our hypothesis that decreased CX3CL1 mediated high adhesive, invasive and survival abilities of *C. albicans* to LKAFs. Increased CX3CL1 secretion partly restored the defect. This suggests that LKAFs have a higher risk of *C. albicans* infection because their antifungal capacity is impaired. We found that oral fibroblasts are an important host defense factor in *C. albicans*-induced infections, where the decreased production and secretion of CX3CL1 may contribute to susceptibility to *C. albicans*.

There are two types of CX3CL1: membrane-bound and soluble for secretion. Membrane-bound CX3CL1 is shown to promote adhesion to leukocytes. The soluble domain of CX3CL1 is known to be chemotactic for NK cells, T cells, monocytes, and mast cells (Wojdasiewicz et al., 2014). The chemotactic effect of CX3CL1 is mediated by the interaction with the specific receptor, CX3CR1 (Imai et al., 1997). Previous research has shown that CX3CR1 dysfunction increases susceptibility of mice and humans to systemic candidiasis (Lionakis et al., 2013). CX3CR1 is important in systemic candidiasis. However, a new research mentioned that CX3CR1 is dispensable for host defense against mucosal candidiasis in mice and humans (Break et al., 2015). Except for the interaction with CX3CR1, we found that CX3CL1 possibly acted as an antifungal cytokine in mucosal candidiasis, independently of the CX3CR1a receptor.

### ERK MAPK and ADAM17 influenced secretion or production of CX3CL1

The extracellular chemokine domain of CX3CL1, which is cleaved by a metalloprotease sheddase, has a significant antifungal activity against *C. albicans* (Takakura et al., 2003). CX3CL1 secretion is possibly mediated via two steps: production and shedding for secretion. A previous study showed that ERK MAPK is involved in CX3CL1 gene transcription and secretion (Uchida et al., 2013). In the present study, we tested the involvement of candidate signaling pathways: ERK, p38, JNK MAPK and NFκB, where the results demonstrated that ERK MAPK was the relevant signaling pathway involved in *C. albicans*-induced CX3CL1 secretion. The p-ERK levels in LKAFs and NFs under *C. albicans* treatment also verified our results. Decreased levels of p-ERK were detected, which indicated that ERK MAPK mediated CX3CL1 secretion in oral mucosal fibroblasts (Figure 5).

In addition, the shedding enzymes ADAM10 and ADAM17 are involved in the cleavage of membrane-anchored cytokines to release soluble CX3CL1. We found LKAFs exhibited decreased mRNA expression of ADAM17 compared with NFs, according to the real-time PCR results (Figure 5). Lower level of ADAM17 may also be a reason for CX3CL1 secretion in LKAFs. Our results confirmed a similar mechanism in previous report that ERK pathway and sheddases regulate CX3CL1 release (Uchida et al., 2013).

However, we are aware that the difference between LKAFs and NFs needs to be confirmed *in vivo* and more *C. albicans* strains would be employed in the future.

In conclusion, LKAFs are more susceptible to *C. albicans* adhesion and invasion than NFs. The secretion of CX3CL1 by fibroblasts contributes to cell resistance to *C. albicans*. Compared with NFs, LKAFs produce less CX3CL1 due to inhibition of the ERK signaling pathway. Decreased ADAM17 may be another possible reason.

## Author contributions

HZ designed the study. DL, RC, XS, QG, CW, XL, YL executed the experiment. RC and DL analyzed the data. RC, DL wrote the manuscript. DL, HZ made critical revision.

## Funding

The study was supported by grants from the National Natural Science Foundation of China (Nos 81172581 and 81272962).

### Conflict of interest statement

The authors declare that the research was conducted in the absence of any commercial or financial relationships that could be construed as a potential conflict of interest.
