# Supplementary material for: Reduced CX3CL1 Secretion Contributes to the Susceptibility of Oral Leukoplakia-Associated Fibroblasts to Candida albicans
Source: Front Cell Infect Microbiol. 2016 Nov 11;6:150. doi: 10.3389/fcimb.2016.00150 (PMC5104956; doi:10.3389/fcimb.2016.00150)
Supplement: Supplementary file 1 [file Table1.DOCX]

**Supplement Table 1.** Patient Information

| No. | Gender | Age | Clinical diagnosis | Pathological diagnosis |
| --- | --- | --- | --- | --- |
| Patient 1 | Male | 43 | OLK | Epithelial hyperplasia |
| Patient 2 | Male | 40 | OLK | Epithelial hyperplasia |
| Patient 3 | Male | 57 | OLK | Epithelial hyperplasia |
| Patient 4 | Female | 58 | OLK | Epithelial hyperplasia |
| Patient 5 | Male | 58 | OLK | Epithelial mild dysplasia |
| Patient 6 | Female | 68 | OLK | Epithelial mild dysplasia |
| Patient 7 | Female | 59 | OLK | Epithelial mild dysplasia |
| Patient 8 | Male | 58 | OLK | Epithelial moderate dysplasia |
| Patient 9 | Female | 37 | OLK | Epithelial moderate dysplasia |
| Patient 10 | Female | 68 | OLK | Epithelial moderate dysplasia |
| Patient 11 | Female | 42 | OLK | Epithelial moderate dysplasia |
| Patient 12 | Male | 45 | OLK | Epithelial moderate dysplasia |
| Patient 13 | Female | 40 | OLK | Epithelial severe dysplasia |
| Patient 14 | Female | 65 | OLK | Epithelial severe dysplasia |
| Patient 15 | Female | 47 | OLK | Epithelial severe dysplasia |
| Patient 16 | Female | 35 | OLK | Epithelial severe dysplasia |
| Patient 17 | Female | 60 | OLK | Epithelial severe dysplasia |
| Patient 18 | Male | 60 | OSCC | High differentiation |
| Patient 19 | Male | 58 | OSCC | High differentiation |

Ages were recorded at the time of biopsy. Diagnoses were made in accordance with clinical and histopathological criteria. OLK: oral leukoplakia; OSCC: oral squamous cell carcinoma.
