# Supplementary material for: Reduced CX3CL1 Secretion Contributes to the Susceptibility of Oral Leukoplakia-Associated Fibroblasts to Candida albicans
Source: Front Cell Infect Microbiol. 2016 Nov 11;6:150. doi: 10.3389/fcimb.2016.00150 (PMC5104956; doi:10.3389/fcimb.2016.00150)
Supplement: Supplementary file 2 [file Table2.DOCX]

**Supplement Table 2.** Primers used for real-time PCR

|  | Forward： | Reverse： |
| --- | --- | --- |
| CX3CL1 | GCTGAGGAACCCATCCAT | GAGGCTCTGGTAGGTGAACA |
| ADAM10 | TCCACAGCCCATTCAGCAA | GCGTCTCAGTGGTCCCATTTG |
| ADAM17 | GAAGAAGTGCCAGGAGGCGATT | CGGGCACTCACTGCTATTACCT |
| β-actin | ATTGCCGACAGGATGCAGAA | GCTGATCCACATCTGCTGGAA |
